# Racial and Ethnic Disparities in Pain Management for Nursing Home Residents: A Scoping Review

**DOI:** 10.1093/geroni/igab046.3199

**Published:** 2021-12-17

**Authors:** Cassandra Dictus, Youngmin Cho, Tamara Baker, Anna Beeber

**Affiliations:** 1 School of Nursing, University of North Carolina - Chapel Hill, North Carolina, United States; 2 University of North Carolina at Chapel Hill, Chapel Hill, North Carolina, United States; 3 University of North Carolina, Chapel Hill School of Medicine, University of North Carolina at Chapel Hill (School of Medicine), North Carolina, United States; 4 University of North Carolina at Chapel Hill, UNC Chapel Hill, North Carolina, United States

## Abstract

Within nursing homes, residents commonly experience pain that unfortunately goes underrecognized and undertreated, having a dramatic negative impact on residents' quality of life. Nursing homes are becoming more racially and ethnically diverse, and there is concerning evidence documenting disparities in the quality of nursing home care. In other healthcare settings, people of diverse race groups often receive less optimal pain management, but the evidence regarding racial disparities has not been synthesized for nursing homes. Thus, the purpose of this review was to investigate what is known about racial disparities related to pain management (e.g. assessment, treatment, preferences) in US nursing homes. We completed a scoping literature review using PRISMA-ScR guidelines and searching PubMed, CINHAL, and Scopus for peer-reviewed, empirical studies. Most studies were older large retrospective cohort studies of administrative data documenting that White residents were more likely than residents of diverse race groups to have pain documented and treated. Only a few studies looked at possible reasons to explain the disparities; differences were not found to be related to nursing staff racial bias nor differences in pain-related diagnoses. However, there was evidence of racial differences in resident behavior and attitudes related to pain management. None of the studies examined systemic factors related to differences among nursing homes, which has been implicated in studies looking at other outcomes including COVID-19. More research is needed which examines the causal mechanisms behind the documented racial disparities in pain management so that gaps in care can be reduced.

